# Supplementary material for: Fracture Healing in 37 Dogs and Cats with Implant Failure after Surgery (2013–2018)
Source: Animals (Basel). 2023 May 5;13(9):1549. doi: 10.3390/ani13091549 (PMC10177280; doi:10.3390/ani13091549)
Supplement: Supplementary file 1 [file animals-13-01549-s001.zip › animals-2327271-Supplementary Table S1.pdf]

Table S1: Area moment of inertia for AO/ASIF plates

Implant	Breadth [b] × Height [h] (mm)	Area moment of inertia [I] (mm4)	Suitable screw sizes (mm)
1.3mm titanium orbital rim plate	3.3 × 0.5	0.03	1.3
1.5mm straight plate	3.8 × 0.8	0.16	1.5
1.5mm LCP	4.25 × 1.0	0.35	1.5
1.5 (2.0) Cuttable plate with spaces (VI)	5.0 × 1.5	1.4	1.5, 2.0
1.5 (2.0) mm Veterinary mini T plate	5.0 × 1.5	1.4	1.5, 2.0
1.5 (2.0) mm Cut-To-Length plate (Synthes)	7.0 × 1.0	0.58	1.5, 2.0
2.0mm Straight Plate	5.0 × 1.0	0.45	1.5, 2.0
2.0 mm DCP (4–6 hole)	5.0 × 1.0	0.42	2.0
2.0 mm DCP (6–8 hole)	5.0 × 1.5	1.4	2.0
1.5 (2.0) mm LC-DCP or LCP (4–7 holes)	5.5 × 1.2	0.79	1.5, 2.0
1.5 (2.0) mm LC-DCP or LCP (6–14 holes)	5.5 × 1.5	1.55	1.5, 2.0
2.0 (2.7) mm Cut-To-Length plate	7.0 × 1.5	2.0	2.0, 2.4, 2.7
2.0 (2.4) mm LC-DCP or LCP (4–8 holes)	6.5 × 1.7	2.66	2.0, 2.4
2.0 (2.4) mm LC-DCP or LCP (8–14 holes)	6.5 × 2.0	4.33	2.0, 2.4
2.7 mm DCP (4–6 hole)	8.0 × 2.0	5.3	2.7
2.7 mm DCP (7–12 hole)	8.0 × 2.5	10.4	2.7
2.7 mm Reconstruction plate	5.0 × 2.7	8.2	2.7
2.7 mm LC-DCP or LCP	7.5 × 2.6	10.99	2.7
2.7 (3.5) mm Veterinary T plate	9.2 × 2.0	6.1	2.7, 3.5, 4.0*
3.5 mm LC-DCP or LCP	11 × 3.3	32.94	3.5, 4.0*
3.5 mm Broad DCP	12.0 × 3.6	46.7	3.5, 4.0*
3.5 mm Broad LC-DCP or LCP	13.5 × 4.2	83.35	3.5, 4.0*
3.5 mm Reconstruction plate	6.0 × 2.8	11.0	3.5, 4.0*
3.5 mm prebent Reconstruction plate	6.0 × 3.6	23.3	3.5, 4.0*
4.5 mm Reconstruction plate	7.0 × 2.8	12.8	4.5, 5.5, 6.5*
4.5 mm T plate	17.0 × 2.0	11.3	4.5, 5.5, 6.5*
4.5 mm Narrow DCP (2–11 hole)	12.0 × 3.8	54.8	4.5
4.5mm Broad DCP (6–26 hole)	16.0 × 4.8	147.5	4.5
4.5 mm narrow LC-DCP or LCP	13.5 × 4.2	83.35	4.0#,4.5, 5.0#, 5.5, 6.5*
4.5 mm Broad LC-DCP or LCP	17.5 × 5.2	205.05	4.0#,4.5, 5.0#, 5.5, 6.5*
4.5 mm Narrow limb lengthening plate (8 hole)	12 × 3.8	54.9	4.5, 6.5* in end holes
4.5 mm Broad limb lengthening plate (8–10 hole)	16 × 4.8	147.5	4.5, 6.5* in end holes
5.5 mm Broad LCP	17.5 × 6.0	315.0	4.0#,4.5, 5.0#, 5.5, 6.5*

Note: AO/ASIF – Arbeitsgemeinschaft für Osteosynthesefragen/Association for the Study of Internal Fixation. Area moment of inertia $I=bh^3/12$ (b = plate dimension parallel to axis around which moment area of inertia is being calculated, h = plate dimension parallel to the applied bending load). The calculations assume a perfect rectangular cross section for plates. DCP, dynamic compression plate; LC-DCP, limited contact dynamic compression plate; LCP, locking compression plate; VI, Veterinary Instrumentation. *Cancellous screws, #locking screws.
